# Green consumers’ behavioral intention and loyalty to use mobile organic food delivery applications: the role of social supports, sustainability perceptions, and religious consciousness

**DOI:** 10.1007/s10668-023-03284-z

**Published:** 2023-05-29

**Authors:** Md. Mahedi Hasan, Md. Al Amin, Md. Shamsul Arefin, Tanjim Mostafa

**Affiliations:** 1 Department of Accounting and Information Systems, Jashore University of Science and Technology, Jashore, 7408 Bangladesh; 2 Department of Marketing, Bangabandhu Sheikh Mujibur Rahman Science and Technology University, Gopalganj, Bangladesh; 3 School of Business and Management, Queen Mary University of London, London, England UK; 4 Department of Management Studies, Bangabandhu Sheikh Mujibur Rahman Science and Technology University, Gopalganj, Bangladesh

**Keywords:** Environmental sustainability, Emotional support, Informational support, Religious values, Trust

## Abstract

Consumer behavior in the food industry has undergone significant changes in recent years, largely driven by growing consumer awareness of environmental, technological, religious, and social concerns. As a result, organic food has emerged as a popular alternative to conventionally produced food. Many emerging nations, including Bangladesh, promote its consumption due to its perceived health and safety benefits. Despite this growing trend, there remains a need for more understanding of consumer behavior, particularly concerning their motivations for continuous purchases toward mobile organic food delivery applications. In order to fill this knowledge gap, this study looks at how six indirect predictors (emotional support, informational support, environmental consciousness, religious consciousness, trust, and technological consciousness) affect customer loyalty through the intention to use organic food. This study employed a purposive sampling technique (i.e., judgmental sampling) and collected data from 386 respondents across three cities in Bangladesh. Data analysis was conducted using SmartPLS 3 software. The study found that all predictors, except for technological consciousness, significantly influenced behavioral intention, which, in turn, significantly influenced loyalty. Additionally, the study revealed that the five predictors, excluding technological consciousness, indirectly influenced loyalty through behavioral intention. The results of this study add to the existing literature on organic food by extending social support theory to include consumers' primary motivations, such as environmental, religious, technological, and social consciousness, as predictors of loyalty to use mobile organic food delivery applications. The study highlights the importance of sustainable food consumption in promoting environmental protection, ensuring social justice, creating economic success, and providing valuable insights for implementers looking to expand the organic food market.

**Graphical abstract:**

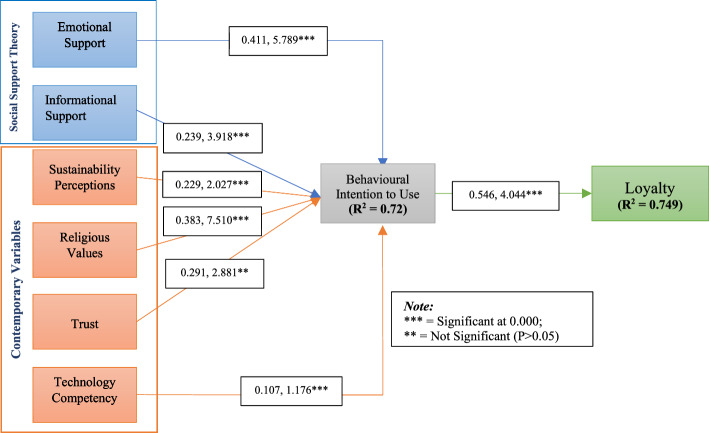

## Introduction

Over the last two decades, there has been a rise in green consumption behavior due to changing climates, consumer health concerns, food preferences, animal welfare, and environmental awareness. As a result, consumers are increasingly motivated to purchase green or organic food because it is produced without artificial chemicals, growth hormones, fertilizers, toxicants, antibodies, or synthetic manure during production and processing (Basha et al., 2015). Several studies also examined the determinants of organic food purchase intention (Khan et al., 2022; Liang & Lim, 2020; Nosi et al., 2020; Pandey et al., 2019; Singh & Alok, 2022; Watanabe et al., 2020) and organic food buying behavior (e.g., Dangi et al., 2020; Hansmann et al., 2020; Talwar et al., 2021; Tandon et al., 2020). Similarly, previous research also primarily focused on personal, social, cultural, and psychological factors (Najib et al., 2022) as well as consumption values (Kushwah et al., 2019a, 2019b; Tandon et al., 2021), but these studies mainly centered on either organic food in general or specific food products. As the organic food industry continues to expand, it is essential to identify the factors that drive green consumers to buy organic products using mobile applications.

Green or sustainable consumption behavior is attractive to religious customers because it is consistent with their beliefs and values (Fischer, 2011). According to a survey by the Pew Research Center (2020), about three-quarters of adults globally identify with a religious affiliation, and about a third consider religion very important in their lives. Similarly, in the USA, data from the General Social Survey (2018) showed that about two-thirds of Americans consider religion an important part of their daily lives. According to a survey conducted by the World Values Survey (2019), most Bangladeshis consider religion an important part of their daily lives, as over 90% of the Bangladeshi population is Muslim. However, little is known about how consumers' religious consciousness influences their organic food buying behavior.

Moreover, besides spiritual principles, consumers are purchasing organic food for different causes, such as the nutritional value of food, several health concerns, taste, and environmental consequences (Hansmann et al., 2020; Nguyen et al., 2019a, 2019b; Talwar et al., 2021), trust (Müller & Gaus, 2015), ecological and political value (Nosi et al., 2020; Tandon et al., 2021), consumption values (Watanabe et al., 2020), and other (e.g., technological concerns such as mobile organic food delivery applications, MOFDA). Here, the MOFDAs are smartphone-based applications used to connect with grocery stores and search for and order organic foods without any physical interaction, lessening the possibility of contamination diseases (Al Amin et al., 2021; Al Amin et al., 2020). Although mobile-based food delivery applications are generic for all types of food, this study focused on the applications that concentrate on organic food. Thus, there has been a growing demand for organic food in Bangladesh since customers started recognizing that food product choices affect their health. Despite the favorable attitudes of consumers toward sustainable food, it remains significant to explore why green consumption is promoted among sustainability-conscious customers.

In addition, environmental consciousness (i.e., environmental sustainability) has intensified the consumer anxiety related to foods they are habituated to consume (Miller & Cassady, 2012) during the outbreaks (e.g., COVID-19, mad cow disease, bird flu, Belgian dioxin scandal, and melamine). Consequently, consumers prefer to consume environment-friendly food products which are organically produced and created without using traditional pesticides, fertilizers with artificial elements, bioengineering, or ionizing radiation, as environmental sustainability are necessary to keep the ecological balance which is a prerequisite for human welfare (Van Loo et al., 2010). The growing concern about sustainable food consumption propels green consumers toward consuming organic foods and products (Verain et al., 2015; Vermeir et al., 2020). Previous research identified that personal beliefs, values, attitudes, behavioral control, subjective norms, trust, environmental concerns, and aspirations are the critical considerations for pro-environmental and organic food behavior (e.g., Al Mamun et al., 2018; Hansmann et al., 2020; Müller & Gaus, 2015). There has been little research on environmental sustainability antecedents of organic food behavioral intention (Nosi et al., 2020; Qasim et al., 2019). Therefore, it is imperative to identify whether environmental sustainability influences consumers purchasing organic foods.

Furthermore, customers expect support for values (i.e., informational and emotional support) to get involved with green food purchasing behavior as a sustainable solution since sustainable consumption may work against environmental degradation and shield against unexpected future economic issues (e.g., Kashif et al., 2021). Previous research primarily focused on the Theory of Reasoned Action, Theory of Planned Behavior, Theory of Consumption Values, Perceived Value Theory, stimuli-organism-response model, and self-determination theory in explaining behavioral intention to purchase organic food. However, social support theory (SST) might explain the underlying reasons regarding organic food purchase intention. In the context of MOFDA, consumers search for reviews of experienced customers, collect information on organic food, make purchase decisions, and provide feedback on their purchase on the online platform (Baek et al., 2012). Users of an online platform such as MOFDA not only exchange knowledge that aids in problem-solving and decision-making but also convey feelings through messages of empathy and support (Al Amin et al., 2021). According to the social support theory, people feel supported by their communities when they receive care and assistance from their peers. Users' perceptions of assistance may have a beneficial effect on their sense of competence. Thus, this study focused on informational and emotional support, which might influence green consumers to purchase organic foods. Moreover, compared to Western countries, very few studies have been conducted in Asian countries on organic food consumption (Li et al., 2020). Notably, there needs to be more research into organic food delivery apps in Bangladesh. Based on this knowledge gap, the current study investigates the factors influencing consumers' intention to use and their continued use of organic food delivery apps in Bangladesh.

The current study aims to contribute to the extent of literature in four ways. First, this study contributes to the social support theory by integrating consumers' primary motivators (i.e., sustainability, social support, trust, religious values, and technological competence) in the organic food context. No prior study focused on the social support theory in the context of mobile-based organic food delivery applications. Second, the present study is one of the pioneer empirical studies that emphasized religious principles to guide customers in choosing foods according to their spiritual values. This study might also guide religious Bangladeshi consumers in their food-purchasing behavior. Third, environmental sustainability can be promoted along with technology consciousness, which might facilitate green consumers' intention to use technology. This study focused on sustainability and technology consciousness in the context of MOFDA. Fourth, previous research on organic food primarily focused on Western countries. This research sheds light on the effects of social support, sustainability, religiosity, and technology competency on organic food purchase intention and loyalty in Bangladesh, an emerging economy.

The following sections comprise a comprehensive analysis of the rest of the study, including a review of related literature, conceptual framework, hypotheses development, research methodology, results, discussions, theoretical contributions, and practical implications. In the concluding section, the study's limitations and potential areas for further research are discussed.

## Literature review and theoretical background

### Organic food

Organic food is referred to as green innovation or ethical innovation by which food products are produced naturally without using any kinds of artificial chemicals, fertilizers, toxicants, antibodies, chemicals, and other synthetic manure (Thøgersen & Zhou, 2012). The U.S. Department of Agriculture defines organic food as "a labeling term that indicates that the food or other agricultural product has been produced through approved methods. These methods integrate cultural, biological, and mechanical practices that foster recycling of resources, promote ecological balance, and converse biodiversity" (Chowdhury et al., 2021). Scholars suggest that organic food has environmental and societal advantages in addition to individual benefits (Cerjak et al., 2010). Previously governed by supply, organic food is now determined by market demand since it has been relaunched as a "new product" under the green, ecological, sustainable, or ethical market segments (Thøgersen, 2010).

Over the last decades, many studies examined the relevant factors and issues on organic food consumption behavior, summarized in Table 1. These incorporate the role of personal, social, cultural and psychological factors (Najib et al., 2022), normative triggers and motivators (Khan et al., 2022), environmental concern (e.g., Hassan et al., 2023; Hansmann et al., 2020; Koklic et al., 2019; Le-Anh & Nguyen-To, 2020), health and food safety concern (e.g., Talwar et al., 2021; Hansmann et al., 2020; Liang & Lim, 2020; Pandey et al., 2019; Sreen et al., 2021; Talwar et al., 2021; Tandon et al., 2021), purchase intentions (e.g., Liang & Lim, 2020; Pandey et al., 2019; Singh & Alok, 2022; Talwar et al., 2021; Watanabe et al., 2020), profiling of organic buyers (Hansen et al., 2018; Nandi et al., 2016), motivations for buying organic food (Hansen et al., 2018; Petrescu et al., 2017; Scalvedi & Saba, 2018; Sobhanifard, 2018), consumer willingness to pay premium (Hasselbach & Roosen, 2015; Lim et al., 2014), ecological and political value (Nosi et al., 2020; Tandon et al., 2021), subjective norms (Dangi et al., 2020; Aitken et al., 2020), and consumer attitudes toward consumptions (Aitken et al., 2020; Dangi et al., 2020; Liang & Lim, 2020; Tandon et al., 2020).

**Table 1:** List of relevant organic food literature

Sl No.	Authors name and year	Context	Model	Sample size	Respondent	Factors (independent)	Factors (dependent)	Significant result
1	Najib et al. (2022)	Organic food	N/A	527	Middle income class	Social, Cultural, Personality and Psychological factors, Attitude toward organic food	Purchase Intention	*Direct predictors of Attitude toward organic food:* Social factors ( +), Cultural factors ( +), Personality factors ( +), Psychological factors ( +) *Direct predictors of Purchase Intention:* Attitude toward organic food ( +), Personality factors (-), Psychological factors (-), Social factors (-), Cultural factors (-)
2	Khan et al. (2022)	Organic food	goal-framing theory	467	Consumers	normative triggers, construct knowledge, motivations	Intentions	All the motivational factors were found significant and positive to consumers' intentions toward organic food. Moreover, normative triggers also influence intentions. The construct knowledge was not found in a direct relationship with intentions; however, a moderating role was established between gain motivations and intentions
3	Le and Nguyen (2022)	Organic food	Theory of Planned Behavior	611	Vietnamese Organic food Consumers	Environmental Awareness, Knowledge of OF, Social Norms, Attitude toward OF Purchase, Personal Norms, Perceived Behavioral Control	Organic food Purchase Intention	*Direct predictors of Attitude toward Purchase:* Environmental Awareness ( +), Knowledge of OF ( +) *Direct predictors of Organic food Purchase Intention:* Knowledge of OF ( +), Attitude toward OF Purchase ( +), Perceived Behavioral Control ( +), Personal Norms ( +), Social Norms ( +) *Direct predictors of Personal Norms:* Social Norms ( +) *Demographic Information:* Male VS Female
4	Miftari et al. (2022)	Organic Food	N/A	300	Urban Consumers in Kosovo	Health Concern, Environmental Concern, Label of origin, Certification as a credence	Attitude toward organic food	*Direct predictors of Attitude toward organic food:* Health Concern ( +), Environmental Concern ( +), Label of origin (-), Certification as a credence ( +) *Demographic Information:* Male VS Female
5	Roseira et al. (2022)	Organic Food	Theory of Planned Behavior	468 Norway, 448 Portugal	Young Consumers	Collectivism, Attitude, Subjective Norms, Perceived price, Availability, Health Consciousness, Environmental Concern, Intention,	Behavior	*Direct predictors of Behavior:* Intention ( +) *Direct predictors of Intention:* Attitude ( +), Subjective Norms ( +), Perceived price ( +), Availability (-), Health Consciousness ( +), Environmental Concern ( +) *Direct predictors of Attitude:* Collectivism ( +) *Direct predictors of Subjective Norms:* Collectivism ( +) *Direct predictors of Perceived price:* Collectivism ( +) *Direct predictors of Availability:* Collectivism ( +) *Direct predictors of Health Consciousness:* Collectivism (-) *Direct predictors of Environmental Concern:* Collectivism ( +) *Demographic Information:* Male VS Female
6	Zayed et al. (2022)	Organic Food	Theory of Planned Behavior	363	Egyptian Consumers	e-WOM, Attitude, Subjective norms, Perceived behavioral control, Environmental Concern, Health Consciousness	Purchase Intention	*Direct predictors of Purchase Intention:* e-WOM (-), Attitude ( +), Subjective norms (-), Perceived behavioral control (-), Environmental Concern ( +), health Consciousness (-) *Direct predictors of Attitude:* e-WOM ( +) *Direct predictors of Subjective norms:* e-WOM ( +) *Direct predictors of Perceived behavioral control:* e-WOM ( +) *Direct predictors of Environmental Concern:* e-WOM ( +) *Direct predictors of health Consciousness:* e-WOM ( +) *Demographic Information:* Male VS Female
7	Dorce et al. (2021)	Organic Vegetables	Theory of Planned Behavior	504	Brazilian Consumers	Perceived health benefits, Perceived sustainability benefits, Attitude, Subjective norms, Perceived behavioral control, Intention, Perceived price	Behavior	*Direct predictors of Behavior:* Intention ( +), Perceived behavioral control ( +), Intention (when Perceived price lower) ( +) *Direct predictors of Intention:* Attitude ( +), Subjective norms ( +) Perceived behavioral control ( +) *Indirect predictors of Behavior:* Attitude through Intention ( +), Subjective norms through Intention ( +), Perceived behavioral control through Intention ( +) *Direct predictors of Attitude:* Perceived health benefits ( +), Perceived sustainability benefits ( +) *Indirect predictors of Attitude:* Perceived health benefits through Attitude ( +), Perceived sustainability benefits through Attitude ( +) *Demographic Information:* Male VS Female
8	Nguyen et al. (2021)	Organic Meat	Theory of Planned Behavior	402	Vietnamese consumers aged over 18 years	Environmental concern, Subjective norm, Attitude toward purchasing organic food, Perceived monetary barriers, Guilt	Purchase Intention	*Direct predictors of Attitude toward purchasing organic food:* Environmental concern ( +), Subjective norm ( +) *Direct predictors of Perceived monetary barriers:* Environmental concern ( +) *Direct predictors of Purchase Intention:* Subjective norm (-), Attitude toward purchasing organic food ( +), Perceived monetary barriers ( +), Guilt ( +) *Demographic Information:* Male VS Female
9	Radulescu et al. (2021)	Organic Fruits and Vegetables	N/A	268	Romanian Organic Fruits and Vegetables Consumers	Consumer understanding of the characteristics of the organic fruits and vegetables, Personal needs and motivations, External influences, Attitude toward organic fruits and Vegetables, Purchase Barriers	Intention to buy Organic Fruits and Vegetables	*Direct predictors of Personal needs and motivations:* Consumer understanding of the characteristics of the organic fruits and vegetables ( +) *Direct predictors of Attitude toward organic fruits and Vegetables:* Consumer understanding of the characteristics of the organic fruits and vegetables ( +), Personal needs and motivations ( +), External influences ( +), Purchase Barriers (-) *Direct predictors of Intention to buy Organic Fruits and Vegetables:* Attitude toward organic fruits and Vegetables ( +), External influences ( +), Purchase Barriers ( +) *Demographic Information:* Male VS Female
10	Talwar et al. (2021)	Organic food	SLT and SOR	928	Japanese customers	Health and food safety concern, Self and ethical self-identity, Openness to change, Willingness to purchase	Buying behavior	*Direct predictors of Openness to change:* Health consciousness ( +), Food consciousness ( +) *Direct predictors of Self-identity*: Health consciousness ( +), Food consciousness ( +) *Direct predictors of Ethical Self-identity:* Health consciousness ( +), Food consciousness ( +) *Direct predictors of Willingness to purchase:* Openness to change ( +), Self-identity (-), Ethical Self-identity ( +) *Direct predictors of Stated Buying behavior:* Willingness to purchase ( +) *Moderating role of Buying Frequency:* Between Openness to change and Willingness to purchase (-), Between Self-identity and Willingness to purchase ( +), Between Ethical Self-identity and Willingness to purchase (-), Between Willingness to purchase and Buying behavior ( +) *Demographic Information:* Male VS Female
11	Hansmann et al. (2020)	Organic food	TPB	620	Swiss households	Attitude, Justification, Health, Trust with label standard, Education, Environmental concern, Income	Organic food purchase	*Direct predictors of Organic food purchase:* Attitude, subjective norms and perceived behavioral control (-), Justification ( +), Environmental concern ( +), Trust with label standard (-), Income ( +), Education ( +), Dynamic variable recent increase in organic share ( +) *Demographic Information:* Male VS Female
12	Tandon et al. (2020)	Organic food	SDT	378	Organic food buyers	Intrinsic and Extrinsic motivation, Attitude, Environmental concern, Trust	Buying behavior	*Direct predictors of Buying behavior:* Attitude (-), Intrinsic motivation (-), Integrated Regulation ( +), Introjected regulation (-), External regulation ( +) *Direct predictors of Attitude:* Intrinsic motivation ( +), Integrated Regulation ( +), Introjected regulation (-), External regulation ( +) *Moderating role of Environmental concern:* Between Intrinsic and Extrinsic motivation and Buying behavior (-) *Moderating role of Trust:* Between Intrinsic and Extrinsic motivation and Buying behavior ( +) *Demographic Information:* Male VS Female
13	Dangi. et al. (2020)	Organic food	TPB	1711	Online based consumers	Attitude, Subjective norms, Perceived behavior control	Purchase intention	*Direct predictors of Purchase intention:* Attitude ( +), Subjective norms ( +), Perceived behavior control ( +) *Direct predictors of Behavior:* Intention ( +) *Demographic Information:* Male VS Female
14	Aitken et al. (2020)	Organic food	RAA	1052	New Zealand Consumers	Perceived behavioral control, Attitude, Subjective norms, Labelling is Actionable, Intention	Purchase Behavior	*Direct predictors of Intention:* Attitude ( +), Subjective norms ( +), Perceived behavioral control ( +) *Direct predictors of Behavior:* Intention ( +) *Direct predictors of Perceived behavioral control:* Labelling is Actionable ( +), Satisfaction with labelling ( +) *Direct predictors of Purchase Behavior:* Perceived behavioral control ( +) *Demographic Information:* Male VS Female
15	Watanabe et al. (2020)	Organic food	PV	274	Brazilian consumers	Functional, Social, Emotional, Economical Value, and Trust	Purchase Intention	*Direct predictors of Purchase intention:* Trust (-), Functional Value ( +), Social Value (-), Emotional Value ( +), Economical Value (-) *Demographic Information:* Male VS Female
16	Liang and Lim (2020)	Organic food	S.O.R. Model	592	Taiwan customers	Attitude, Labeling and trust toward organic food, Nutritional value, Environmental protection effects, Health concern, Natural food preference, Health risk	Purchase intention	*Direct predictors of Attitude toward organic food:* Nutritional value (-), Environmental protection effects ( +), Attitude toward organic food labeling ( +) *Direct predictors of Attitude toward organic food labeling:* Nutritional value ( +), Environmental protection effects ( +), *Direct predictors of Trust in organic food labeling:* Nutritional value ( +), Environmental protection effects ( +), Attitude toward organic food labeling ( +) *Direct predictors of Purchase intention:* Health concern ( +), Natural food preference ( +), Health risk ( +), Attitude ( +), Attitude toward organic food labeling (-), Trust ( +) *Demographic Information:* Male VS Female
17	Nosi et al. (2020)	Organic food	TRA	158	Tuscan University students	Ecological and Political value, Corporate Social responsibility image, Attitude	Behavioral intention	*Direct predictors of Attitude:* Ecological Welfare ( +), Political value (-), CSR Image ( +) *Direct predictors of Behavioral intention:* Attitude ( +) *Demographic Information:* Male VS Female
18	Pandey et al. (2019)	Organic food	TPB	200	Organic customers	Revealed information by organic food items, Subjective norms, Perceived information, Attitude, Trust	Purchase intention	*Direct predictors of Trust:* Revealed information by organic food items ( +), Subjective norms ( +), Perceived information ( +), *Direct predictors of Purchase intention:* Trust ( +), Attitude ( +), Revealed information by organic food items ( +), Subjective norms ( +), Perceived information ( +) *Demographic Information:* Male VS Female
19	Koklic et al. (2019)	Organic food	T.R.A. and TPB	462	Adult Customers	Past organic food consumption, Attitude toward organic food consumption, Personal norms, Environmental concern	Organic food buying intention	*Direct predictors of Attitude toward organic food* *Consumption:* Past organic food consumption ( +) *Direct predictors of Organic food buying intention:* Attitude toward organic food consumption ( +), Past organic food consumption ( +), Personal norms ( +) *Direct predictors of Personal norms:* Past organic food Consumption ( +), Environmental concern ( +) *Demographic Information:* Male VS Female
20	Sreen et al. (2021)	Natural products	BRT	949	Consumers of natural products	Health consciousness, Reasons for, Reasons against, Attitude, Environmental Concern	Brand Love	*Direct predictors of Brand Love:* Attitude ( +), Reasons for ( +), Reasons against (-) *Indirect predictors of Brand Love:* Reasons for through Attitude ( +), Reasons against through Attitude ( +) *Direct predictors of Attitude:* Reasons for ( +), Reasons against ( +), Health consciousness ( +), *Indirect predictors of Attitude:* Health consciousness through Reasons for ( +), Health consciousness through Reasons against (-), *Direct predictors of Reasons for consuming natural products:* Health consciousness ( +) *Direct predictors of Reasons against consuming natural products:* Health consciousness (-) *Moderating role of Environmental Concern:* Between Reasons for and Brand Love ( +), Between against for and Brand Love ( +), Attitude and Brand Love (-) *Moderating role of Household Size:* Between Reasons for and Brand Love (-), Between against for and Brand Love (-), Attitude and Brand Love (-), *Demographic Information:* Male VS Female
21	Talwar et al. (2021)	Organic food	SLT and SOR	928	Multiple cities people	Health consciousness, Food safety concern, Openness to change, Self-identity, Ethical self-identity, Willing to purchase	Stated Buying Behavior	*Direct predictors of Openness to change:* Health consciousness ( +), Food safety concern ( +) *Direct predictors of Self-identity:* Health consciousness ( +), Food safety concern (-), *Direct predictors of Ethical self-identity:* Health consciousness ( +) Food safety concern ( +) *Direct predictors of Willing to purchase:* Openness to change ( +), Self-identity (-), Ethical self-identity ( +) *Direct predictors of Stated Buying Behavior:* Willing to purchase ( +) *Moderating role of Buying Frequency:* Between Openness to change and Willing to purchase (-); Between Self-identity and Willing to purchase ( +); Between Ethical self-identity and Willing to purchase (-); Between Willing to purchase and Stated Buying Behavior ( +) *Demographic Information:* Male VS Female
22	Le-Anh and Nguyen-To (2020)	Organic food	TPB	129	Organic food buyers	Awareness of organic food, Information on organic food, Food safety concern, Environment concern, Perceived barriers, Attitude toward organic food, Perceived value,	Purchasing intention	*Direct predictors of Attitude toward organic food:* Awareness of organic food ( +), Information on organic food (-), Food safety concern ( +), Environment concern (-), Perceived barriers (-), Perceived value ( +) *Direct predictors of Awareness of organic food:* Information on organic food ( +) *Direct predictors of Purchasing intention:* Attitude toward organic food ( +), Perceived value ( +) *Demographic Information:* Male VS Female
23	Tadnon et al. (2021)	Organic food	IRT and DFT	928	Japan Multiple cities people	Health consciousness, Ecological welfare, Nutritional content, Natural content, Value barrier, Usage barrier, Risk barrier	Stated Buying Behavior (SBB)	*Direct predictors of Ecological welfare:* Health consciousness ( +) *Direct predictors of Nutritional content:* Health consciousness ( +) *Direct predictors of Natural content:* Health consciousness ( +) *Direct predictors of Value barrier:* Health consciousness ( +) *Direct predictors of Usage barrier:* Health consciousness ( +) *Direct predictors of Risk barrier:* Health consciousness ( +) *Direct predictors of Stated Buying Behavior:* Ecological welfare ( +), Nutritional content ( +), Natural content (-), Value barrier ( +), Usage barrier (-), Risk barrier (-) *Moderating role of Buying Involvement:* Between Ecological welfare and SBB (-); Between Nutritional content and SBB ( +); Between Natural content and SBB (-); Between Value barrier and SBB (-), Between Usage barrier (-), Between Risk barrier ( +) *Moderating role of Gender:* Between Ecological welfare and SBB ( +); Between Nutritional content and SBB ( +); Between Natural content and SBB ( +); Between Value barrier and SBB (-), Between Usage barrier (-), Between Risk barrier ( +) *Demographic Information:* Male VS Female
24	Tandon et al. (2020)	Organic food	Self-determination theory	378	Shopping mall customers	Intrinsic Motivation, Extrinsic Motivation, Introjected regulation, Integrated regulation, External regulation, Environmental concern, Trust, Attitude,	Buying Behavior (BB)	*Direct predictors of Buying Behavior:* Attitude (-), Intrinsic Motivation (-), Integrated regulation ( +), Introjected regulation ( +), External regulation ( +) *Direct predictors of Attitude:* Intrinsic Motivation ( +), Integrated regulation ( +), Introjected regulation ( +), External regulation ( +) *Moderating role of Environmental concern:* Between Intrinsic Motivation and BB (-), Between Integrated regulation and BB (-), Between Introjected regulation and BB ( +), Between External regulation and BB (-) *Moderating role of Trust:* Between Intrinsic Motivation and BB (-), Between Integrated regulation and BB (-), Between Introjected regulation and BB (-), Between External regulation and BB (-) *Demographic Information:* Male VS Female
25	Nguyen et al. (2019a)	Organic food	TPB, VBN, ABC	572	Younger and Educated Vietnamese consumers	Health consciousness, Environmental concern, Organic-label trust, Traditional-self, Modern-self, Attitude, Subjective norm, Perceived-behavioral control	Purchase Intention	*Direct predictors of Purchase Intention:* Attitude ( +), Subjective norm ( +), Perceived-behavioral control ( +), *Direct predictors of Attitude:* Subjective norm ( +), Health consciousness ( +), Environmental concern (-), Organic-label trust ( +), Traditional-self ( +), Modern-self ( +) *Demographic Information:* Male VS Female
26	Nguyen et al. (2019b)	Organic food	TPB	609	Vietnamese citizens	Environmental concern, Food safety concern, Health consciousness, Organic food knowledge, Attitudes toward buying organic food, Green marketing, Price barriers	Organic food purchase behavior	*Direct predictors of Attitudes toward buying organic food:* Environmental concern ( +), Food safety concern ( +), Health consciousness ( +), Organic food knowledge ( +) *Direct predictors of Organic food purchase behavior:* Attitudes toward buying organic food (-), Green marketing ( +), Price barriers ( +) *Demographic Information:* Male VS Female
27	Kushwah et al., (2019a, 2019b)	Organic food	IRT	452	Buyers- Nonbuyers of organic food	Image barrier, Value barrier, Risk barrier, Purchase intention, Ethical consumption intention	Choice behavior	*Direct predictors of Choice behavior:* Image barrier (-), Value barrier (-), Risk barrier (-), Purchase intention ( +), Ethical consumption intention ( +) *Direct predictors of Purchase intention:* Image barrier (-), Value barrier ( +), Risk barrier (-), Ethical consumption intention ( +) *Direct predictors of Ethical consumption intention:* Risk barrier (-), Image barrier (-), Value barrier ( +) *Demographic Information:* Male VS Female
28	Pham et al. (2018)	Organic food	TRA and TPB	289	Graduate and Postgraduate students	Environmental concern, Food safety concern, Health consciousness, Food taste, Media expose, Perceived barriers, Attitude toward organic food	Purchase intention	*Direct predictors of Attitude toward organic food:* Environmental concern (-), Food safety concern ( +), Health consciousness 3, Food taste (-), Media expose ( +), Perceived barriers ( +) *Direct predictors of Purchase intention:* Perceived barriers ( +), Attitude toward organic food ( +) *Demographic Information:* Male VS Female
29	Hansen et al. (2018)	Organic food	SCT	1176	Danish food Consumers	Environmental consciousness, Health consciousness, social consciousness, Personal values, Organic food identity, Control variables	Intentional organic food behavior	*Direct predictors of Organic food identity:* Environmental consciousness ( +), Health consciousness ( +), Social consciousness ( +), Control variables ( +) *Direct predictors of Intentional organic food behavior:* Organic food identity ( +), Control variables ( +) *Demographic Information:* Male VS Female
30	Ryan and Casidy (2018)	Organic food	BRT	617	Amazon’s Mechanical Turk (MTurk) platform	Values, Brand reputation, Reason for and against consuming organic food, Attitude toward organic food	Purchase intention	*Direct predictors of Reason for consuming organic food:* Values ( +) *Direct predictors of Reason against consuming organic food:* Values ( +) *Direct predictors of Attitude toward organic food:* Values ( +), Reason for consuming organic food ( +), Reason against consuming organic food ( +) *Direct predictors of Purchase intention:* Attitude toward organic food ( +) *Indirect predictors of Attitude toward organic food:* Values through Reason for consuming organic food ( +), Values through Reason against consuming organic food (-) *Moderating role of Brand reputation:* Between Values and Reason for consuming organic food ( +); Between Values and Reason against consuming organic food ( +); Between Attitude toward organic food and Reason for consuming organic food ( +); Between Attitude toward organic food and Reason against consuming organic food ( +) *Demographic Information:* Male VS Female
31	Konuk (2017)	Organic food	Equity Theory	349	Consumer in Sakarya	Price Fairness, Organic food satisfaction, Trust in organic food	Purchase intentions	*Direct predictors of Organic food satisfaction:* Price Fairness ( +) *Direct predictors of Trust in organic food:* Price Fairness ( +), Organic food satisfaction ( +) *Direct predictors of Purchase intentions:* Price Fairness ( +), Organic food satisfaction ( +), Trust in organic food ( +) *Demographic Information:* Male VS Female

TPB = Theory of Planned Behavior, VBN = value-belief-norm, ABC = the attitude-behavior-context, IRT = Innovation Resistance Theory, BRT = Behavioral Reasoning Theory, DFT = & Dual- Factor Theory, SCT = Social Comparison Theory

Moreover, organic food research was primarily conducted in developed countries, such as the USA (Lee & Goudeau, 2014; Rodman et al., 2014; Ryan & Casidy, 2018) and Europe (Janssen, 2018; Janssen & Hamm, 2014). In contrast, relatively few empirical studies were conducted in Asia, such as in China (e.g., Chen et al., 2014; Hasimu et al., 2017), Iran (e.g., Asif et al., 2018; Sobhanifard, 2018), Jordon (e.g., Lillywhite et al., 2013), Pakistan (Asif et al., 2018), Vietnam (e.g., Pham et al., 2018), Malaysia (e.g., Chekima et al., 2017; Lim et al., 2014), Thailand (e.g., Sriwaranun et al., 2015), Turkey (e.g., Çabuk et al., 2014), and Taiwan (e.g., Chang & Chang, 2017). Furthermore, there is a dearth of empirical research on organic food consumption in developing countries like Bangladesh (Kabir & Islam, 2022; Sumi & Kabir, 2018; Zheng et al., 2021). Nonetheless, most of these studies focused on the factors influencing organic food consumers' intentions and choices. In particular, the analogized implications between religious customers' primary motivations and organic food consumers' behavior toward using MOFDA were largely disregarded in these investigations. Consequently, based on the existing literature, we propose the interlink between organic food consumer behavior (i.e., buying intention and customer loyalty) and four primary consumer motivations: (i) environmental consciousness (i.e., sustainability), (ii) social consciousness (i.e., emotional support, and informational support), (iii) religious consciousness (i.e., religious values and trust) and (iv) technological consciousness (technological competence).

### Social support theory

The term "social support" describes an individual's awareness of, or access to, social resources offered by others within their network, whether such resources are formally organized or not (Gottileb & Bergen, 2010). Several scholars argue that social support investigates how people feel cared for regarding the responsiveness and the facilitation of other people within their networks or social groups (Tajvidi et al., 2017; Nadeem et al., 2019). Researchers in sociology, medicine, and even marketing has paid attention to social support's importance (Tajvidi et al., 2017; Hajli et al., 2014; Nadeem et al., 2019). However, recent technological developments and the introduction of MOFDA have made it more critical than ever to gain a deeper comprehension of this phenomenon.

Consumers in an online platform place a premium on the opinions and advice of their peers when making purchases. In the context of MOFDA, users use the online platform to look for reviews written by other customers, gather knowledge on organic food, decide which products to buy and leave comments on those products. In addition, involvement in online settings and the exchange of experiences significantly impact the development of the ideas and choices held by other users of the platform (Baek et al., 2012). For instance, when picking a host through a sharing economy website like Airbnb or Homeaway, a traveler is most likely to look at the remarks left by other members of the platform first.

However, one of the most important customer motivational theories (i.e., SST; Cullen, 1994) was not considered in the extent of green or organic food literature. SST is based on the perception and practicality essential for accepting assistance from the people and, most importantly, their support network. These supportive resources can be emotional (e.g., nurturance), informational (e.g., advice), or instrumental. Moreover, scientists and practitioners from various social, behavioral, medical, and nursing fields have studied the relevance of social support in contributing to health and well-being (Leahy-Warren, 2014). In addition, SST has also been used by many other studies (e.g., Algharabat & Rana, 2020; Hsu et al., 2018; Lee & Chen, 2020; Molinillo et al., 2017; Tajvidi et al., 2017) in the context of online community engagement, social commerce interaction, massive open online course, brand value co-creation, e-commerce site correspondingly. Furthermore, Nadeem et al. (2019) developed an integrated model based on SST and relationship support theory (RST) together with some factors (e.g., privacy, security, non-deception, Reliability, Trust, satisfaction, and commitment) to predict the consumer value co-creation intention. Yusuf et al. (2018) also expanded TRA and SST, incorporating contemporary factors (e.g., information quality, information credibility, website quality, innovativeness, attitude toward eWOM, and eWOM engagement). Accordingly, this study extends SST, including contemporary variables such as environmental sustainability, technology competency, and religious consciousness in MOFDA.

### Conceptual framework

Due to the several efforts of the marketers and social awareness of the government, organic food products have created a niche in the consumer's mind (Hansmann et al., 2020; Sumi & Kabir, 2018). As a result, scholars are increasingly interested in researching organic food products based on well-established theoretical lenses encapsulated in Table 2. For example, Lin et al. (2020) investigated the organic food purchase intention with social commerce intention based on the Theory of Consumption Values (TVC). They found that social commerce characteristics and organic food characteristics both influence functional and emotional value. These values also influence the purchase intention in the context of China's most popular online platform. In another study, Pacho (2020) determined the factors of purchase intention of organic food based on the Theory of Planned Behavior (TPB). He found that attitude, subjective norms, perceived behavioral control, and health consciousness influence the buying decision in the context of middle-class customers in Tanzania. Besides, Talwar et al. (2021) explored the factors of purchasing and stated buying behavior toward organic foods based on the Social Learning Theory (SLT) and Stimulus-Organism-Response Theory (S–O-R) model. They detected that health consciousness, food safety concern, openness to change, self-identity, ethical self-identity, and willingness to purchase influence stated buying behavior in the context of Japan.

**Table 2:** List of theories used in existing organic food literature

Theory	Authors name and year
Theory of Planned Behavior	Hansmann et al. (2020); Dangi et al. (2020); Pandey et al. (2019); Koklic et al. (2019); Pacho (2020); Nagaraj (2020); Le-Anh and Nguyen-To (2020); Nguyen et al. (2019a, 2019b); Nguyen et al. (2019a, 2019b); Pham et al. (2018)
Theory of Consumption Values	Lin et al. (2020); Kushwah et al. (2019a, 2019b); Amin and Tarun (2020); Kashif et al. (2021)
Theory of Reasoned Action	Nosi et al. (2020); Koklic et al. (2010); Pham et al. (2018)
Stimuli-organism-response model	Talwar et al. (2021); Liang and Lim (2020); Talwar et al. (2021)
Social learning theory	Talwar et al. (2021)
Dual factor theory	Tandon et al. (2021)
Behavioral reasoning theory	Sreen et al. (2021); Tandon et al. (2020); Ryan and Casidy (2018)
Innovation resistance theory	Tandon et al. (2021); Kushwah et al. (2019a, 2019b)

Moreover, Kushwah et al., (2019a, 2019b) examined ethical consumption intention and choice behavior based on the Theory of Consumption Values (TVC). They found that social, emotional, conditional, and epistemic values influenced consumption value and choice behavior in the context of community-centric buyers and non-buyers in the USA. Tandon et al. (2021) examined the facilitator and inhibitor factors of stated buying behavior based on the dual-factor and innovation resistance theories. They picturized that facilitators factors with ecological welfare, nutritional content, natural content, and inhibitors factors with value barriers influenced the buying behavior in the context of consumers in Japan. Besides, Nagaraj (2020) illustrated organic food's health and safety consciousness based on the Theory of Planned Behavior (TPB). It was established that food safety concerns, consumer attitude, and health consciousness influenced purchase intention behavior in the context of customers in metropolitan cities in India. Furthermore, the previous organic food studies also utilized several other models, such as the TPB (Hansmann et al., 2020; Dangi & Gupta et al., 2020; Pandey et al., 2019), SDT (Tandon et al., 2020), Perceived Value Theory (PV) (Watanabe et al., 2020), TRA (Nosi et al., 2020), and S–O–R (Liang & Lim, 2020).

**Fig. 1:**
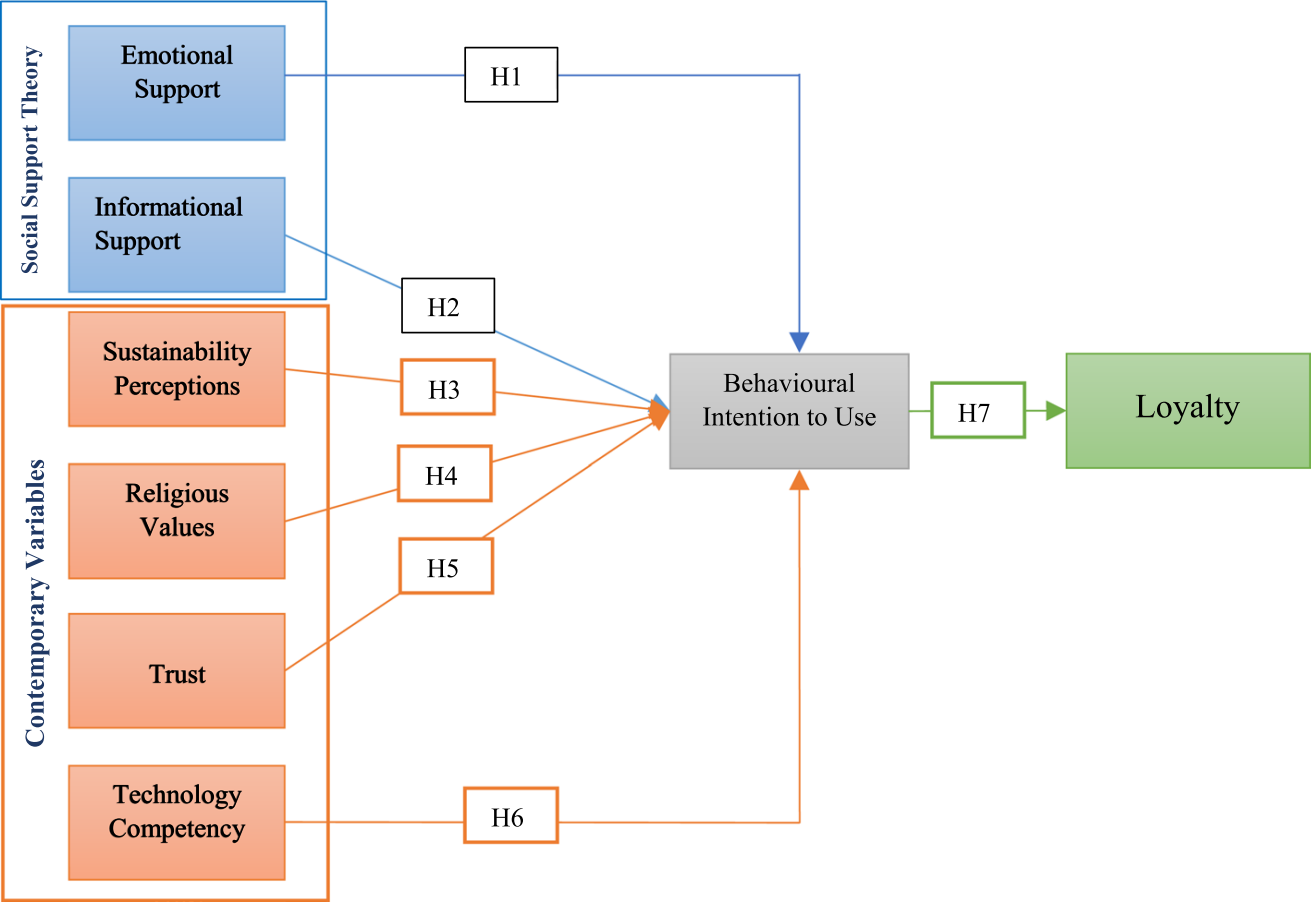
Conceptual framework of the study

Consequently, after a careful evaluation of relevant models and theories, we developed and validated a unique theoretical framework based on SST to explore the impact of consumer primary motivational factors (i.e., social support, sustainability, trust, religious values, technological competence) on behavioral intention and loyalty to use MOFDAs. However, we have excluded instrumental and appraisal support from SST since these two variables are primarily linked to reducing the likelihood of delinquency and crime, and we argue that these are less likely to determine the consumer motive to purchase organic food following the previous research (Sheikh et al., 2019; Lin et al., 2018). Other motivational factors, such as technological competency, religious values, trust, and sustainability, are predicted to influence users' buying behavior. Technology competency stands for the consumer abilities, imperative skills, and knowledge essential to use a particular technology (e.g., MOFDA) (Cutshall et al., 2021), whereas religious values contribute as an instigator between attitude and purchase intention (Memon et al., 2019). Likewise, trust encourages consumer decision-making in a complex food market. By achieving consumers' trust (i.e., firm belief in the reliability, truth, or ability of someone or something), the marketer can retain long-term customer relationships (Hamzaoui-Essoussi et al., 2013). Furthermore, sustainability perceptions instigate customers to inspire environmental awareness and purchase ecologically benign and non-harmful products (Michaelidou & Hassan, 2008). D'Amico et al. (2016) identified environmental awareness or sustainability as one of the most important determinants of consumer organic food behavior. Figure 1 depicts the structural relationship of the proposed theoretical model, which is illustrated in the following sections.

### Social supports

Individuals' perceptions of being cared for, receiving responses, and receiving assistance from members of their social groups are measured by the idea of social support in sociology (Liang et al., 2011). Human beings need social interactions to satisfy their needs for belonging and support in determining the role of social commerce (Liang et al., 2011). Among the four types of social support (i.e., emotional, instrumental, informational, and appraisal support), two, such informational and emotional support, are essential for social engagement in information system usage. In particular, they are the most effective support mechanisms influencing a user's behavior toward using a particular technology (Liang et al., 2011; Sheikh et al., 2019; Lin et al., 2018). In the context of organic food, Ashraf (2021) identified that perceived social support does not influence organic food purchase intention. However, in that study, he did not examine the role of two important supports (emotional and informational) in purchasing organic foods.

#### Emotional support (EST)

"Emotional support" is commonly used to describe showing sympathy, concern, affection, understanding, or encouragement to another person (Zhao et al., 2019). High levels of perceived emotional support are associated with people's feelings of being cared for and accepted within the group (Yang, 2021). When people in a group give each other emotional support, it lowers their stress and passively helps them resolve the problem (Zhao et al., 2019). The relationships between group members could strengthen with this kind of support (Hu et al., 2019). Individuals may have less anxiety due to the emotional support they receive in the online community (Sheikh et al., 2019). Customers' emotional and mental well-being can be improved when they receive support and encouragement from friends when shopping online (Lin et al., 2018). Consumers' trust, commitment, and satisfaction are expected to rise due to the emotional support provided in online networks (Hajli, 2014). Previous research primarily focused on emotional support in social commerce (e.g., Hajli, 2014; Lin et al., 2018; Sheikh et al., 2019; Yang, 2021). Accordingly, online consumers are emotionally supported by their social members, such as friends and family, toward finding and purchasing organic foods. Thus, we can posit the following hypothesis:

##### H1

Emotional support positively influences behavioral intention to use MOFDAs.

#### Information support (IST)

Informational support helps individuals solve problems, create new ideas, or make good decisions by providing advice, guidance, or helpful information (Chen & Shen, 2015). Some other studies also found that informational support influences social commerce intention (e.g., Lee et al., 2020; Molinillo et al., 2017; Riaz et al., 2020) in the context of social commerce social networking. Besides, informational support indirectly influences purchase intention (Bazi et al., 2019; Yusuf et al., 2018) in the context of co-creation and purchase intention in social commerce. Hence, IST might influence BIU by giving information and guidelines to create new ideas and knowledge regarding organic food products. Therefore, the following hypothesis can be predicted:

##### H2

Information support positively influences behavioral intention to use MOFDAs.

### Environmental consciousness

Sustainability perception is "an individual's judgment or evaluation of an environmental issue or an event based on personal experiences and attitudes toward certain environmental conditions" (Lin et al., 2021, p. 12,225). The environmental or sustainability concern related to organic food refers to food production without using substances and activities which may damage the environment (Lamonaca et al., 2022). The existing research found that sustainability perception directly influences behavioral intention to use in the context of sustainability and social media communication, product aesthetics, and organic food (Kong et al., 2020; Wang & Hsu, 2019; Sriyogi et al., 2016). More importantly, unsustainable or harmful products to the environment ultimately lead to environmental degradation, which is dangerous to an individual's balanced life. Hence, sustainability perception (SP) may influence BIU toward organic food to maintain the environment and human well-being. Hence, we can posit the following hypothesis:

#### H3

Sustainability perception positively influences behavioral intention to use MOFDAs.

### Religious consciousness

Religious consciousness refers to belief in God and adherence to the principles established by religious leaders—consisting of standards, judgment, and trust held by individuals (Usman et al., 2017). In addition, religious consciousness (i.e., religious values and trust) reforms the customers' daily lives, consumption practices, purchasing attitudes, and behavior. According to Wilkes et al. (1986), customers with higher religiosity prefer local brands known as 'deshi foods' or 'organic food' in Bangladesh. Consumer perceptions of halal food show that consumers link halal, its nature, and processing methodology (which includes ingredients, handling, and the employment of diverse technologies) with cleanliness and food safety from start to finish (Mutmainah, 2018). Moreover, every religion has its food consumption guidelines (Pratiwi, 2018). For instance, beef is restricted to the consumer by Hinduism, whereas pork is prohibited in Islam and Judaism (Awan et al., 2015). In addition, most religions guide consuming foods only if they are prepared, processed, stored, packaged, handled, and transported hygienically (Pratiwi, 2018). Many previous empirical studies also showed a strong influence of religion on consumer behavior (Bailey & Sood, 1993; Hirschman, 1985; Kamaruddin, 2007). Besides, organic food is considered 'safe food,' which is argued to be allowable to eat while maintaining religious values (Liu et al., 2013; Pratiwi, 2018). Similarly, we argue that Bangladeshi customers committed to their religion are sensitive to the dietary guidelines for accepting food products as per their spiritual values. Therefore, exploring how religious values influence customers' acceptance of mobile organic food purchasing behavior has been essential. In this study, the individual's spiritual consciousness mainly discusses the religious values and trust that may influence organic food consumers' behavior.

#### Religious values (RV)

Religion is a set of ideas, indications, symbols, and practices that enable people to feel near to God and serve as a guide for their interpersonal relationships. Religious values or promises play a moderating role between attitude and purchase intention (Memon et al., 2019) and stimulate behaviors and attitudes when the role of expectations (faith laws of religion, e.g., foods habit) from religion are internalized (Weaver & Agle, 2002). Moreover, a previous study identified that religious values directly influence behavioral intention to use in the context of Islamic bank trust and halal purchase intention (Suhartanto, 2019; Memon et al., 2019). Hence, religious values may influence BIU to use MOFDAs since these values provide a prominent role in shaping individuals' perceptions and intentions. Therefore, the following hypothesis can be predicted:

##### H4

Religious values positively influence behavioral intention to use MOFDAs.

#### Trust (TRT)

According to Trust and Commitment Theory (Morgan & Hunt, 1994), trust is defined as when one party trusts the reliability and integrity of the other parties. In other words, individuals' trust is their belief that the other party will meet their needs in the future (Suhartanto, 2019). Trust is essential in encouraging decision-making in a complex food market (Hamzaoui-Essoussi et al., 2013). Accordingly, trust is formed when both parties (i.e., consumers and sellers) have the assurance to launch honest and trustworthy relationships regarding product quality and payment guarantee. Prior research found that trust directly influences consumers' behavioral intention to use in the context of food delivery apps, mobile banking, information technology, and organic food (Muangmee et al., 2021; Troise et al., 2020; Merhi et al., 2019; Suhartanto, 2019; Kabra et al., 2017; Müller & Gaus, 2015; Bashir & Madhavaiah, 2015). Accordingly, the trust may influence BIU to reduce risks while using MOFDAs. Therefore, we can predict the following hypothesis:

##### H5

Trust positively influences behavioral intention to use MOFDAs.

### Technological consciousness (TC)

Technological consciousness or technology competency (TC) is the degree to which an individual has the essential skills and knowledge to use technology (Cutshall et al., 2020). Several studies found that technology competency indirectly influences behavioral intention to use social commerce in the context of social commerce (e.g., Cutshall et al., 2020, 2021). Consumers' proficiency in handling technology may increase their confidence in using technology. Thus, technology competency may influence BIU because of the willingness to recognize new technology, such as easy-to-use online apps. Hence, this study predicts the following hypothesis:

#### H6

Technology competency positively influences behavioral intention to use mobile organic food delivery applications.

### Behavioral intention to use and loyalty

Behavioral intention can be referred to as comparative behavioral measures, price sensitivity, acquisition, and information sharing (Uddin, 2013). Behavioral intention is considered a post-purchase action experience by a consumer after consuming the product or service (Al Amin et al., 2023; Uddin, 2013). The research found that behavioral intention to use directly influences loyalty in the context of social media and electronic household products (Lv et al., 2018; Uddin, 2013). Moreover, some studies found a positive relationship between brand awareness and purchase intention in the context of heuristic information processing, social network, youtube marketing, social media, and core brand attitude (Dabbous & Barakat, 2020; Febriyantoro, 2020; Hutter et al., 2013; Tan et al., 2021; Wu & Lo, 2009). In the context of MOFDA, we argue that the positive user experience of consumers may lead them to purchase organic food repeatedly. Thus, the following hypothesis can be predicted:

#### H7

Behavioral intention to use positively influences loyalty to use MOFDAs.

## Research methodology

### Research design

A mixed methods approach is often recommended to understand better and develop theories around emerging phenomena, such as human-like interactional competencies in mobile based applications and their role in user loyalty through trust, support or behavioral intention. By combining qualitative and quantitative data collection and analysis techniques, researchers can better understand the underlying mechanisms at play (Venkatesh et al., 2013). To ensure the rigor and validity of our study, we followed the mixed methods research guidelines proposed by Venkatesh et al. (2013).

In the qualitative study, we conducted a semi-structured interview with five MOFDA sellers to confirm the results from the qualitative study and, more importantly, to understand and reconcile the counterintuitive findings. This qualitative study aimed to confirm the measurement items’ intelligibility and relevance to the study. This qualitative study identified boundary conditions that helped us contextualize and provide a more comprehensive understanding of the main study’s indispensability. This approach allowed us to gain a more nuanced and holistic understanding of the phenomenon being investigated. Besides, a pilot study with twenty-five frequent users of MOFDA was conducted. According to the feedback from the respondents of interviews and pilot study, we constructed the survey questionnaire with two sections—(i) demographic information and (ii) measurement items, taken from previous studies and primarily written in English. Furthermore, before the main study, we followed the back-translation method through which the questionnaire was translated into Bengali (Brislin, 1976) since the main study was conducted in Bangladesh.

In the quantitative study, we conducted a self-administrated questionnaire survey on the respondents who were above 18 years old and resided in three large cities in Bangladesh (i.e., Dhaka city, Jessore city, and Gopalganj city). We also screened those customers who used or experienced at least one web or mobile-based organic food delivery application (e.g., Food Panda, Chaldal.com, KhaasFood, Shwapno, Agora, Mina Bazar, Organiconline.com.bd, and Naturals.com.bd) over the last six months. Given the unknown nature of the population and sampling frame, our study utilized a non-probability sampling method. This approach allowed us to select respondents based on subjective judgment while adhering to the guidelines outlined in Saunders et al. (2009). We employed purposive sampling, precisely the judgmental sampling method, to address potential issues associated with convenience sampling. Additionally, we aimed to include a diverse range of target respondents to increase the likelihood of representative results. The subsequent section provides detailed overviews of this quantitative study.

### Measurement items

The measurement items were extracted from existing studies and shown in Appendix. We adopted a five-point Likert scale that ranges from 1 (strongly agree) to 5 (strongly disagree). For example, the items of behavioral intention to use with three indicators (e.g*., “I intend to continue using MOFDA to purchase organic food in the future”*) were taken from Venkatesh et al. (2012) and Lin et al. (2018) and the four items of loyalty (e.g., *“I am always loyal toward MOFDA when I get the required values”)* was extracted from Atulkar (2020).

To measure six predictors, a total of 27 measurement items were used: emotional support, with four items (e.g., “*When faced with difficulties on Mobile Organic Food Delivery Applications (MOFDAs) stand on my side with me”* extracted from Liang et al. (2011). Similarly, information support, with three items (e.g., *“Purchasing organic food Using* MOFDAs, *some people would offer suggestions when I needed help”*) was adapted from Nadeem et al. (2019) and Sheikh et al. (2019); technology competency (Cutshall et al., 2020, 2021) was evaluated (e.g., “*I am always interested in purchasing organic food from online*”); sustainability perception was taken from Kianpour et al. (2014) and Kim et al. (2015) consisting of six indicators (e.g., *“The product is friendly to the environment and harmless for nature”*); Trust (Ebrahim, 2019) was measured using four items (e.g., *“MOFDA is honest.”)* and religious values (Lockie et al., 2002; Honkanen et al., 2006) was measured using five items (e.g., “*MOFDA is not forbidden by my religion*). Moreover, our reliability test, shown in Table 4, depicts that Cronbach’s alpha’s values lie between 0.732 and 0.892 which also proves that all required criteria are met and relevant to conduct this study.

### Data collection

In November 2021, data were gathered from respondents in Bangladesh as part of our efforts to test our hypotheses. Prior to administering the questionnaire, we obtained informed consent from all participants. To facilitate the data collection process, we used various virtual platforms, such as Mail, Messenger, and WhatsApp, to send each respondent a cover letter and questionnaire. We allowed participants one week to complete the questionnaire and sent a final appeal to those who failed to respond within the deadline. Our findings were consistent with prior studies (Chatterjee et al., 2002; Dillman, 2000; Hall, 2008), which found no significant difference between online and paper surveys. A total of 640 questionnaires were distributed to our target respondents, with feedback received from 397 participants. After discarding incomplete responses, we analyzed 386, representing 60.31%. An overview of the demographic profiles of the respondents is shown in Table 3.

**Table 3:** Demographic profile of the respondents

Variables	Number	Percentage
*Gender*		
Male	212	54.9
Female	174	45.1
*Age*		
15–20 years	32	8.3
21–25 years	255	66.1
26–30 years	77	19.9
30–35 years	22	5.7
*Occupation*		
Govt. service	6	1.6
Private service	27	7.0
Self-employment	57	14.8
Bank/non-bank fin. institutions	22	5.7
Agro (plant/animals/fisheries)	13	3.4
Garments/textile	12	3.1
Students	184	47.7
Others	65	16.8
*Income level*		
0–10,000	209	54.1
10,000–20,000	56	14.5
20,000–30,000	80	20.7
30,000–40,000	20	5.2
40,000–50,000	8	2.1
Above 50,000	13	3.4
*Education*		
Higher secondary	40	10.4
Graduation	63	16.3
Under graduation	259	67.1
Post-graduation	24	6.2
*MOFDA Platforms *		
Food panda	154	39.9
Chaldal	48	12.4
Shwapno	66	17.1
Meena Bazar	41	10.6
Agora	40	10.4
Organic online	18	4.7
Other	19	4.9

### Statistical analysis

The data were analyzed using structural equation modeling (SEM). SEM can simultaneously measure multiple dependent variables, causal models, or equations (Chin, 1998; Cohen et al., 2018; Wang et al., 2019). Among the two types of SEM, including CB-SEM (covariance-based SEM) and PLS-SEM (partial least square SEM), CB-SEM analyzes the fit of observed variables based on a covariance matrix, whereas the PLS-SEM examines the dependent and independent variables based on prediction and estimates by maximizing the explained variances (Al Amin et al., 2022a; Al Amin et al., 2022b; Wang et al., 2019; Ringle et al., 2015; Akdim et al., 2022). Moreover, the PLS-SEM predicts the extent of variations in endogenous constructs because of exogenous constructs. To analyze confirmatory factor analysis and structural interactions between the research variables, we used PLS-SEM using Smart PLS 3.0 software (Al Amin, 2022; Akdim et al., 2022; Hair et al., 2017; Ringle et al., 2015). The study used a listwise deletion procedure due to its simplicity, generality, and default in SPSS (Allison, 2003) to treat the missing data before formal data analysis. The study also confirmed the data normality through testing skewness and kurtosis. The findings depicted that skewness values were between − 1.825 and + 1.472, while kurtosis values were between − 0.721 and + 1.691. Since these values were within the recommended limit (Kline, 1998) of skewness and kurtosis (skewness: ± 3 and kurtosis: ± 10); thus, the data were normally distributed.

### Common method bias (CMB)

We also controlled common method bias associated with evaluating the independent and dependent variables from the perceptions of the target respondents. The study ensured statistical and actual measures before and after data collection to minimize the possibility of CMB. To determine the CMB, we tested Harman’s one-factor test. According to Podsakoff et al. (2003), the cut-value for the first factor should be less than 50% of the total variance explained. The principal component analysis (PCA) results showed that eight identified factors with more than 1.00 eigenvalues were responsible for 81.40% of the total variance. The first factor was accountable for only 24.91%, within the tolerable limit shown in Table 5.

## Results and discussions

### Measurement model

We tested the measurement model through construct reliability and convergent and discriminant validity.

#### Construct reliability

To examine the construct reliability, we followed the suggestions of Hair et al. (2017). The construct reliability was investigated by assessing the composite reliability (CR), Cronbach’s alpha, and roh_A (cutoff value of CR: > 0.70; α: > 0.7; rhoA: > 0.70). According to the suggestions, the criteria were met for all eight constructs provided in Table 4.

**Table 4:** Construct reliability

Constructs	Items	Loadings	Cronbach’s Alpha	rhoA	CR	AVE
Behavioral intention to use	BIU1	0.834	0.762	0.779	0.817	0.600
	BIU2	0.746				
	BIU3	0.739				
Loyalty	LTY1	0.701	0.827	0.801	0.824	0.610
	LTY2	0.853				
	LTY3	0.782				
Emotional supports	EST1	0.709	0.881	0.857	0.893	0.585
	EST2	0.729				
	EST3	0.871				
	EST4	0.827				
	EST5	0.731				
	EST6	0.705				
Informational supports	IST1	0.728	0.792	0.758	0.779	0.541
	IST2	0.715				
	IST3	0.762				
Technology competency	TC1	0.705	0.732	0.707	0.837	0.508
	TC2	0.719				
	TC3	0.728				
	TC4	0.701				
	TC5	0.709				
Sustainability perceptions	SP1	0.811	0.783	0.781	0.908	0.622
	SP2	0.813				
	SP3	0.772				
	SP4	0.703				
	SP5	0.819				
	SP6	0.809				
Religious values	RV1	0.721	0.892	0.903	0.867	0.566
	RV2	0.808				
	RV3	0.738				
	RV4	0.771				
	RV5	0.720				
Trust	TRT1	0.823	0.851	0.891	0.848	0.584
	TRT2	0.716				
	TRT3	0.772				
	TRT4	0.741				

AVE = Average variance extracted, CR = Composite reliability

#### Convergent validity

Convergent validity was evaluated by testing the Average Variance Extracted (AVE) and factor loading (FL) of the constructs shown in Table 4. This study fulfilled the required criteria for AVE (AVE: > 0.50) and FL (FL: > 0.7) according to the recommendations of Hair et al. (2017) to confirm the model’s construct reliability.

#### Discriminant validity

We also examined the discriminant validity of the measurement model testing the Heterotrait-Monotrait Ratio of Correlations (HTMT), which should be no more than 0.85 (HTMT: < 0.85) (shown in Table 5) and Fronell-Larcker criterion (Hair et al., 2017; Henseler et al., 2015). According to Hair et al. (2017), the off-diagonal elements (correlations among the study constructs) should be less than the diagonal elements (the squared root of AVE) to meet the Fronell–Larcker criterion shown in Table 6. Tables 5 and 6 show that all the required criteria were met for discriminant validity.

**Table 5:** Heterotrait-Monotrait Ratio (HTMT) and result of Exploratory Factor Analysis (EFA)

	BIU	LTY	EST	IST	TC	SP	RV	TRT
BIU	–							
LTY	0.772	–						
EST	0.701	0.832	–					
IST	0.003	0.801	0.382	–				
TC	0.568	0.239	0.520	0.897	–			
SP	0.369	0.675	0.763	0.221	0.091	–		
RV	0.817	0.391	0.540	0.632	0.692	0.760	–	
TRT	0.745	0.551	0.365	0.441	0.259	0.114	0.015	
Eigenvalues	9.41	5.08	3.9	3.25	2.86	2.53	1.98	1.75
% of Variance	24.91%	13.45%	10.32%	8.59%	7.58%	6.69%	5.23%	4.63%
Cumulative %	24.91%	38.36%	48.68%	57.27%	64.85%	71.54%	76.77%	81.40%

**Table 6:** Fronell–Larcker criterion

	BIU	LTY	EST	IST	TC	SP	RV	TRT
BIU	0.775							
LTY	0.641	0.781						
EST	0.682	0.576	0.765					
IST	0.093	0.530	0.087	0.736				
TC	0.193	0.093	0.451	0.533	0.713			
SP	0.327	0.183	0.285	0.109	0.132	0.789		
RV	0.328	0.381	0.623	0.231	0.639	0.291	0.752	
TRT	0.245	0.126	0.087	0.224	0.143	0.441	0.317	0.836

The diagonal element represents the squared root of AVE

### Structural model

According to Hair et al. (2017), the coefficient of determinations (R2), the strength of the effect (f2), and the significance level of the path coefficient validate the structural model. The current research tested all hypotheses through the bootstrap with 5000 resamples and measured t-statistics for testing the path coefficient following the guidelines suggested by Henseler et al. (2016).

#### Coefficient of determinations

The squared multiple correlations are shown in Table 8, where the coefficient of determinations (R^2^) of BIU is 0.720, which states that independent variables (i.e., EST, IST, TC, SP, and RV) cause 72% variation in dependent variables (i.e., BIU). Here, LTY is changed by 74.9% due to changes in BIU.

#### Strength of effect and blindfolding-based cross-validated redundancy

In contrast to the R^2^ value, the strength of effect sizes f^2^ is tested to know the representative influence of different constructs/variables in one single model (Henseler et al., 2015). Chin (1998) and Henseler et al. (2015) suggest that the strength of effect sizes (f^2^) value of 0.02 is a small effect, 0.15 is the medium effect, and 0.35 is a large effect. Table 8 shows that the strength of effect sizes ranged from 0.023 to 3.021.

The present study also confirmed the blindfolding-based cross-validated redundancy (Q^2^), which measures the predictive capability of specified parameters in PLS-SEM. The Q^2^ value greater than zero (0) for a particular endogenous construct indicates the overall path model’s predictive relevance (Hair et al., 2017). The results shown in Table 8 confirmed the required criterion of Q^2^.

#### Model fit summary

**Fig. 2:**
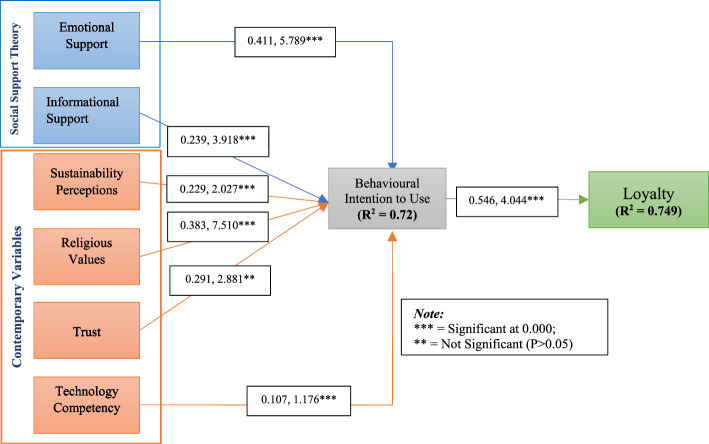
Structural model of the study

The study also examined the model fit indices. The suggested value of SRMR and RMSEA should be less than 0.08 (Browne & Cudeck, 1993), RMS_theta should be less than 0.1 (Hair et al., 2019), CFI, TLI, and NFI should be less than 0.95 (Hu & Bentler, 1999). The results of the model fit indices (shown in Table 7) satisfied the threshold values for both the measurement model and structural model (Hair et al., 2017; Hu & Bentler, 1999; Khanra & Joseph, 2019; Browne & Cudeck, 1993). The structural model of the study is depicted in Fig. 2.

**Table 7:** The results of model fit

Model fit indices	Measurement model	Structural model
CFI	0.964	0.972
TLI	0.954	0.950
RMSEA	0.039	0.036
SRMR	0.077	0.053
RMS_theta	0.092	0.094
NFI	0.962	0.958

SRMR = Standardized root mean square residual; RMSEA = Root mean square error of approximation; CFI = Comparative fit index; TLI = Tucker–Lewis index; NFI = Normative fit index

#### Hypotheses testing

We conducted a routine bootstrapping with five thousand resamples to examine the study's proposed hypotheses and path coefficient shown in Table 8. The results showed that the influence of EST (β = 0.411, *t*-statistics = 5.789, *p* < 0.000) and IST (β = 0.239, *t*-statistics = 3.918, *p* < 0.000) on BIU were positive and significant. According to our assumptions, H1 is supported by the study findings, which are aligned with the previous studies such as in organic food consumption (Kashif et al., 2021), ethical consumption (Kushwah et al., 2019a, 2019b), in green purchase intention (Amin & Tarun, 2020) since the consumer is concerned with emotional support. Similarly, H2 is supported, and this finding is consistent with the existing research findings on social commerce intention and social networks (Lee et al., 2020; Molinillo et al., 2017; Riaz et al., 2020). We argue that since social support (EST and IST) can provide personal warmth and understanding, it can also be thought of as the ability to respond to one’s psychological needs (Molinillo et al., 2017). Such positive experiences may help people meet social interactions by satiating their social relationships with friends. Since internet connections are online and frequently rely on messaging at different virtual communities, social support may assist MOFDA users in purchasing decisions.

**Table 8:** Path coefficient and hypothesis test result

Hypotheses	Relationships	Path coefficient	SD	t-Statistics	*p* Values	VIF	f2	Remarks
H1	EST→ BIU	0.411	0.071	5.789	0.000	1.852	0.312	supported
H2	IST→ BIU	0.239	0.061	3.918	0.000	2.092	0.192	supported
H3	SP→ BIU	0.229	0.113	2.027	0.000	3.009	0.117	Supported
H4	RV→ BIU	0.383	0.051	7.510	0.000	1.885	3.021	Supported
H5	TRT→ BIU	0.291	0.101	2.881	0.000	2.763	0.023	supported
H6	TC→ BIU	0.107	0.091	1.176	0.061	1.995	0.001	Not Supported
H7	BIU→ LTY	0.546	0.135	4.044	0.000	2.481	0.384	supported

Coefficient of determinations (R2), BIU = 0.720, LTY = 0.749; Blindfolding-based cross-validated redundancy (Q2), BIU = 0.537, LTY = 0.618

Moreover, in H3, the influence of SP (β = 0.229, *t*-statistics = 2.027, *p* < 0.000) on BIU was positive. Accordingly, we argue that environmental consciousness motivates consumer behavioral intention to purchase organic food using MFODA. Previous studies supported our hypothesized relationship between environmental consciousness and behavioral intention, which have found that environmental concerns might encourage consumers’ interest and influence their purchase decisions (Kong et al., 2020; Wang & Hsu, 2019). Van Loo et al. (2010) mentioned that consumers prefer to consume environmentally friendly food products that are organically produced. Organic food helps environmental sustainability by prohibiting the usage of traditional pesticides or fertilizers, which is necessary to keep the ecological balance needed for human welfare.

In addition, the results showed that RV (β = 0.383, *t*-statistics = 7.510, *p* < 0.000) and TRT (β = 0.291, *t*-statistics = 2.881, *p* < 0.00) positively and significantly influenced BIU. Thus, our predicted relationships between religious consciousness and consumer intention to consume organic food are consistent with the findings of the previous studies (e.g., Memon et al., 2019; Suhartanto, 2019) in determining trust and halal purchase intention. The existing literature suggests that religious consciousness determines an organized order of religious values, trusts, commitments, ritual beliefs, and practices to empower followers. Hence, the commitment to follow a divine rule, religious values, or trust may impact how they choose and consume products and services and communicate with others.

Furthermore, the influence of TC (β = 0.107, *t*-statistics = 1.176, *p* < 0.061) on BIU to use MOFDA was found insignificant in the study findings in H6, which is inconsistent in the context of m-commerce websites (Agrebi & Jallais, 2015), the use of mobile phones for shopping (Zhang et al., 2012). Thus, modern technological advancements have made users capable of using MOFDA.

Finally, in H7, BIU (β = 0.546, *t*-statistics = 4.044, *p* < 0.000) significantly influenced loyalty in purchasing organic food. Customers having positive service experiences with strong relational and emotional attachments to the service are more likely to purchase repeatedly. Hence, MOFDA service providers must find innovative ways to keep up the continuous usage intention of the respective users with better performance and state-of-the-art technology.

Moreover, we also confirmed and tested the variance inflation factor (VIF) to assess the lateral collinearity effect validating the structural model. We followed the recommendation of Hair et al. (2017) to assess VIF values, which should be at most five signposting lateral multicollinearity issues among the constructs. In addition, they suggested that the ideal value of VIF should be at most 3.00 or very close to 3.00. The study results shown in Table 8 illustrate no multicollinearity problem or VIF issues for this study.

## Contributions

### Theoretical contributions

The current study has four theoretical implications to the extent of the literature.

Firstly, *the role of social support in organic food literature might add to the existing literature* since social support would be inborn for the community members to spread a particular organic food purchasing experience and suggestions as an extension for sharing other supportive information. Moreover, informational support might provide remedies, plans, or interpretations in a virtual setting. In contrast, emotional support focuses on expressing one’s concerns and can thus assist in the indirect resolution of difficulties. There is a dearth of empirical studies examining how social support affects consumers’ propensity to buy organic foods. This study showed that the social support generated through online platforms highly affects consumers' purchase decisions. In contrast to the study by Ashraf (2021), which found no association between social support and organic food purchase intention, this study shows a positive relationship in the context of MOFDAs.

Second, *religious values and trust are essential mechanisms for the continued use and loyalty toward MOFDAs.* In Bangladesh, religious consciousness is a multidimensional subject of several dimensions: values, beliefs, involvement, rituals, and fear of divine punishment. Religious consciousness drives most customers’ motivation to purchase organic food products that allow consumers’ faith laws or religious values. This study identified that consumers with high religious consciousness are more likely to purchase organic food products using MOFDA.

Third, *sustainable perceptions and technology consciousness contribute to users’ behavioral intention and loyalty toward MOFDAs over and above the social support dimensions*. A better understanding of the role of ecological sustainability perceptions is essential since sustainable perception is a way for customers to express their views and the value provided by environmentally friendly goods or services. The complex structure of cognitive and emotional factors appears to provide a strategy for fostering sustainable growth of ecological resources. In contrast, understanding technology consciousness was crucial since Bangladesh is an emerging country in terms of information and communication technology since the sustainability of the uses of MOFDAs is dependent on the capability of customers to use a particular application.

Finally, *this study applies an* *extended social support theory* and provides the first empirical evidence on organic food customers’ post-experience perceptions, emotions, continuance intention, and loyalty in a developing country, which has been disregarded from the mainstream literature.

### Managerial implications

The current baseline research model might guide the policymakers, experts, and practitioners on how they can satisfy the Bangladeshi religious consumers to be loyal to a particular MOFDA. Bangladeshi MOFDA developers and marketers should focus on enhancing MFODA interoperability as a developing economy. Furthermore, these apps should be designed and promoted to shops in a way consistent with customers’ current lifestyles, values, beliefs, behavior, norms, purchasing habits, and individual requirements, wants, and expectations. For example, busy persons, ‘office employees,’ or high-income groups with limited leisure time may be unable to shop in-store to assess product size, shape, functionality, or interoperability.

Moreover, organic food producers, sellers, and distributors should consider environmental protection concerns and pleasing aesthetic experiences. The MOFDA designers will need to create pleasant and consistent user experiences in the future. Simultaneously, marketers must be challenged to determine the most enticing synthesis of the Bangladeshi consumer religious consciousness. Since Bangladesh is a country of active followers of their religions, marketers should be aware of Muslim, Hindu, Christian, and Buddhist’ spiritual principles and practices. For example, marketers should abide by Islamic law’s primary sources, the Quran and Hadith, to sell organic food products to Muslim consumers. In addition, they should be aligned with Hindu laws for managing Hindu consumers. Thus, organic food cultivators and MOFDA providers should consider religiosity a relatively discrete continuum.

In addition, users’ comments and reviews should be scrutinized to ensure they are relevant, credible, and current so that new users see the system as a valuable data resource. Customers are more likely to recommend and accept advice from friends and family members about which MOFDAs to use. As a result, active users should receive additional financial incentives (e.g., price discounts, quantity discounts, and points) and show loyalty. Existing customers are critical in generating income by recruiting and endorsing new customers based on these incentives. Finally, the drive to use and share information would dwindle without such social support. Deciding how to promote the upkeep of such a supportive atmosphere is a critical topic that MOFDA service providers must consider while running their operations. Hence, the social support spectrum should be considered since supportive interactions among group members make them feel closer to one another and more content in exchanging information.

### Limitations and further study

The current study, like most others, has some limitations. First, we have collected the data in Bangladesh, a developing country, which may limit the generalization of study findings into different geographical locations. Hence, future studies can consider respondents from other culturally diverse countries to measure unobserved heterogeneity in the population. Second, because the study was cross-sectional, prone to methodological biases, and causation between variables may be limited. However, a follow-up study with a longitudinal design can confirm the causality of the association across time. Third, as MOFDA is still growing and relatively new to Bangladeshi customers, customer judgments might often vary by a group based on unidentified referents. There is a possibility to exist unobserved subgroups of opinions about MOFDA. Thus, understanding this growing sector and its customers is essential through further research to continuously redefine the service offering and managerial implications.

## Conclusion

This study contributes to the organic food literature by examining the predictors of MOFDA customer post-experience evaluations, social support, religious consciousness, environmental sustainability concern, and intention to use MOFDAs. This study is one of the pioneer studies to integrate the Social Support Theory with four contemporary variables: (i) environmental consciousness (i.e., sustainability), (ii) social consciousness (i.e., emotional support and informational support), (iii) religious consciousness (i.e., religious values and trust) and (iv) technological consciousness (technological competence) according to the suggestions of existing literature. The role played by these variables provides a direction for marketers and researchers to build trust in environmental consciousness, social support, religious values, and technological consciousness with MOFDAs. In addition, this study tackled this important topic in Bangladesh, which represents a non-western, emerging economy that has yet to be noticed in mainstream literature.

## Data Availability

The datasets generated during and/or analyzed during the current study are available from the corresponding author on reasonable request.

## Appendix

**Table:** Measurement Items

Constructs and Source	Measurement items
*Emotional support*	
Liang et al. (2011)	EST1: When faced with the difficulties with Mobile Organic Food Delivery Applications (MOFDAs) some people are on my side with me
	EST2: When faced with difficulties with MOFDAs, some people comforted and encouraged me
	EST3: When faced with difficulties with MOFDAs, some people listened to me and talk about my private feelings
	EST4: When faced with difficulties with MOFDAs, some people expressed interest and concerned in my well-being
*Information support*	
Nadeem et al. (2019)	IST1. Purchasing organic food using MOFDA, some people would offer suggestions when I needed help
Sheikh et al. (2018)	IST2. Consumer’s ethical perceptions of organic food are consistent MOFDAs
Liang et al. (2011)	IST3. People who use MOFDA, help me discover causes and provide suggestions when needed
*Technology competency*	
Cutshall et al. (2020, 2021)	TC1: I am always interested in purchasing through MOFDAs
	TC2: I enjoy purchasing organic food through MOFDAs
	TC3: I use MOFDAs every day for purchasing organic food
	TC4: I am not afraid to use MOFDAs to purchase organic food
	TC5: My ability to learn new technology (e.g., MOFDAs) is high
*Trust*	
Ebrahim (2019)	TRT1: This company is honest
	TRT2: This company stands for my happiness
	TRT3: This company works hard to satisfy me
	TRT4: This company’s promises are real
*Sustainability perception*	
Kianpour et al. (2014) Kim et al. (2015)	SP1: The product purchased through MOFDAs is friendly to environment and harmless for nature
	SP2: The product purchased through MOFDAs has environmental certification for saving energy
	SP3: The product purchased through MOFDAs is green and harmless for human
	SP4: The MOFDA seller offers green delivery service
	SP5: The MOFDA seller uses recycled packing materials for delivery
	SP6: The MOFDA seller invests for the environment
*Religious values*	
Lockie et al. (2002) Honkanen et al. (2006) Worthington et al. (2003)	RV1: Purchasing through MOFDA is not forbidden by my religion
	RV2: Purchasing through MOFDA is in harmony with my religious views
	RV3: My religious beliefs lie behind my whole approach to life
	RV4: Religious beliefs influence all my dealings (e.g., dealings with purchasing through MOFDA) in life
	RV5: Religion is especially important to me because it answers many questions about the meaning of life when I use MOFDA
*Behavior intention to use*	
Venkatesh et al. (2013) and Lin et al. (2019)	BIU1: I intend to continue using MOFDAs in the future
	BIU2: I will always try to use MOFDAs in purchasing organic food
	BIU3: I plan to continue to use MOFDAs frequently
*Loyalty*	
Atulkar (2020)	LTY1: I am always loyal toward MOFDAs when I get the required values
	LTY2: Positive perception and satisfaction influence me to take repurchase decisions using MOFDAs
	LTY3: I am always loyal toward MOFDAs which creates emotional attachment
